# Dissecting the Signaling Events That Impact Classical Nuclear Import and Target Nuclear Transport Factors

**DOI:** 10.1371/journal.pone.0008420

**Published:** 2009-12-24

**Authors:** Mohamed Kodiha, Dan Tran, Andreea Morogan, Cynthia Qian, Ursula Stochaj

**Affiliations:** Department of Physiology, McGill University, Montreal, Canada; Texas Tech University Health Sciences Center, United States of America

## Abstract

**Background:**

Signaling through MEK→ERK1/2 and PI3 kinases is implicated in many aspects of cell physiology, including the survival of oxidant exposure. Oxidants play a role in numerous physiological and pathophysiological processes, many of which rely on transport in and out of the nucleus. However, how oxidative stress impacts nuclear trafficking is not well defined.

**Methodology/Principal Findings:**

To better understand the effect of stress on nucleocytoplasmic trafficking, we exposed cells to the oxidant diethyl maleate. This treatment activated MEK→ERK1/2 as well as PI3 kinase→Akt cascades and triggered the inhibition of classical nuclear import. To define the molecular mechanisms that regulate nuclear transport, we examined whether MEK and PI3 kinase signaling affected the localization of key transport factors. Using recently developed tools for image acquisition and analysis, the subcellular distributions of importin-α, CAS, and nucleoporins Nup153 and Nup88 were quantified in different cellular compartments. These studies identified specific profiles for the localization of transport factors in the nucleus and cytoplasm, and at the nuclear envelope. Our results demonstrate that MEK and PI3 kinase signaling as well as oxidative stress control nuclear trafficking and the localization of transport components. Furthermore, stress not only induced changes in transport factor distribution, but also upregulated post-translational modification of transport factors. Our results are consistent with the idea that the phosphorylation of importin-α, CAS, Nup153, and Nup88, and the O-GlcNAc modification of Nup153 increase when cells are exposed to oxidant.

**Conclusions/Significance:**

Our studies defined the complex regulation of classical nuclear import and identified key transport factors that are targeted by stress, MEK, and PI3 kinase signaling.

## Introduction

Elevated levels of reactive oxygen species play a major role in human disease by contributing to type 2 diabetes, ischemia/reperfusion damage, cardiovascular diseases, stroke, Alzheimer's disease as well as numerous neurodegenerative disorders and syndromes [1]–[7]. In response to oxidative stress, cells activate multiple signaling cascades, including the PI3 kinase→Akt/PKB and MEK→ERK1/2 pathways. Moreover, crosstalk between PI3 kinase and MEK→ERK1/2 signaling cascades has been described in different model systems [8]–[11]. Activation of PI3 kinase and MEK induces a large number of downstream events that occur both in the nuclear and cytoplasmic compartment [12]; however, the impact of signaling on nuclear transport is only beginning to emerge.

Macromolecular trafficking across the nuclear envelope is mediated by nuclear pore complexes (NPCs), and for most cargos it relies on a specific transport apparatus. In particular, members of the importin-α and β families are crucial to move proteins in and out of the nucleus [13], [14]. Classical nuclear import is one of the major routes to deliver proteins to the nucleus. This pathway requires the dimeric carrier importin-α/β1, for which importin-α serves as an adaptor that links the cargo to importin-β1. For delivery to the nucleus, the cargo initially binds to importin-α/β1 in the cytoplasm, thereby generating a trimeric import complex which then moves across the NPC. Once inside the nucleus, the import complex dissociates, whereupon importin-α and importin-β1 return separately to the cytoplasm. Importin-α recycling to the cytoplasm requires CAS (cellular apoptosis susceptibility protein), a carrier of the importin-β family [15]. Aside from its direct role in nuclear transport, CAS is also implicated in cell proliferation, apoptosis and the control of p53-mediated gene expression [16], [17].

In addition to carriers and adaptors like importin-α, nucleoporins, also called nups, are essential to move cargoes across the nuclear envelope. Nucleoporins contribute to different aspects of nuclear trafficking; for instance, nucleoporins with FG repeats provide docking sites for import complexes during their translocation across the NPC. Some nucleoporins are stably bound to NPCs, whereas others are mobile and play a more dynamic role in trafficking [18]. Nup153 is such a mobile nucleoporin which contains multiple copies of FG repeats. Under normal growth conditions, Nup153 predominantly locates to the nuclear side of the NPC where it participates in transport of protein and RNA [19]. By contrast, the nucleoporin Nup88 is a structural component of cytoplasmic NPC filaments, but was recently shown to have additional functions inside the nucleus [20]–[22].

Publications from several groups have demonstrated that classical nuclear import is sensitive to various forms of stress [21], [23]–[27]. However, despite the increasing body of data that connects nuclear transport inhibition to stress, the molecular mechanisms and signaling events that underlie the stress-induced changes in nuclear trafficking are poorly understood. To gain a better understanding of these events, we exposed human culture cells to the oxidant diethyl maleate (DEM), a compound that depletes intracellular glutathione. Our results demonstrate that the activities of two different signaling routes, the MEK→ERK and PI3 kinase pathways, correlated with the localization of transport components, both upon oxidative stress and under nonstress conditions. In addition, we provide evidence that oxidative stress not only changes the intracellular distribution of transport factors, but also alters their post-translational modification.

## Results

For the studies described here we have exposed HeLa cells to mild stress in order to limit the irreversible damage of cellular functions. We have previously shown that under these conditions nuclear envelopes remain intact; moreover, most of the cells remain viable and will recover from this treatment [21]. Our research further suggested that oxidative stress targets several factors that are crucial for nuclear protein transport. To date, only a limited number of quantitative studies have been undertaken to define the impact of stress and signaling events on nuclear import. Such quantitative analyses, however, will provide a better understanding of how changes in cell physiology regulate intracellular trafficking. In addition, quantitative studies are necessary to reliably identify small but significant changes that can not easily be substantiated with qualitative approaches. Towards this aim, we have now performed a systematic investigation under normal and stress conditions of classical nuclear import and key factors that are essential for nuclear trafficking. Our research reveals how oxidant exposure and signaling events control classical nuclear import and target multiple components of the nuclear transport apparatus.

### Classical Nuclear Import of the Reporter Protein NLS-mCherry Is Inhibited by Oxidative Stress

**Figure 1:**
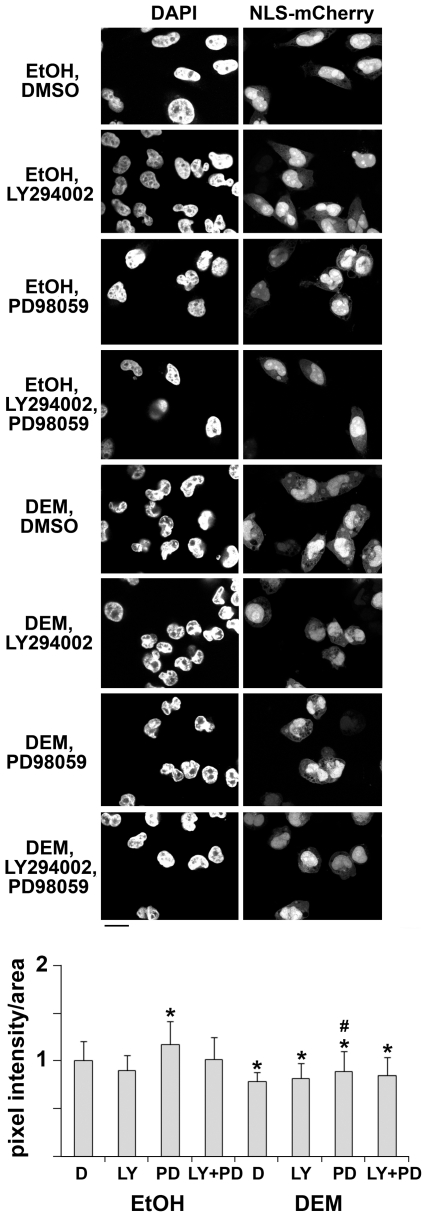
Stress and pharmacological kinase inhibitors modulate classical nuclear import. HeLa cells were transiently transfected with plasmids encoding NLS-mCherry and pre-incubated for 1 h with DMSO (D), 50 µM LY294002 (LY), 25 µM PD98059 (PD) or a combination of both drugs. After pre-incubation, cells were treated with ethanol (EtOH, controls) or 2 mM diethyl maleate (DEM, oxidative stress) for 4 hours at 37°C. DMSO or inhibitors were also present throughout the incubation with ethanol or DEM. The localization of NLS-mCherry was monitored by confocal microscopy under the different conditions shown in the figure. Size bar is 20 µm. Pixel intensities in nuclear and cytoplasmic compartments were quantified using the MetaXpress multi wavelength cell scoring module; for each condition the distribution of NLS-mCherry was measured for at least 50 cells. Results are shown as pixel intensity/area. One-way ANOVA was used to identify significant differences. Comparisons were made among all groups with DMSO-treated unstressed cells (D, EtOH) as reference; * shows *P*<0.05. For comparisons among all stressed cells DMSO/DEM samples were used as a reference; # indicates *P*<0.05.

Many of the endogenous cargos for classical nuclear import interact with other cellular components, either in the nucleus, the cytoplasm or both. Such interactions may anchor the cargo in different cellular locations, thereby affecting its nucleocytoplasmic distribution. To avoid this complication, we generated the reporter NLS-mCherry, a small fluorescent protein that contains SV40 nuclear localization sequence (NLS) fused to the monomeric protein mCherry. The small size of NLS-mCherry allows diffusion across the NPC, whereas nuclear accumulation of the reporter protein relies on active nuclear import. Thus, the nuclear/cytoplasmic (nuc/cyt) distribution of NLS-mCherry serves as an indicator of classical import activity. Using tools recently developed for image acquisition and quantification of fluorescence in different cellular compartments [28], we measured the nuc/cyt ratio of NLS-mCherry under different experimental conditions. In brief, confocal microscopy combined with quantitative image analysis was applied to determine the concentration of NLS-mCherry inside the nucleus and in the cytoplasm. The nuc/cyt ratio obtained for nonstress conditions was defined as 1; this ratio will decrease if classical import is compromised. Fig. 1 demonstrates that DEM treatment reduced significantly the nuc/cyt ratio for NLS-mCherry (compare DMSO, denoted D, for ethanol-treated controls with DMSO for DEM-stressed cells). These results are consistent with the idea that classical import is inhibited when cells are exposed to oxidant.

### MEK Signaling Controls Classical Nuclear Import under Normal and Stress Conditions

MEK and PI3 kinase signaling cascades are essential components that regulate cell survival; this includes signaling events triggered by various physiological or pathological changes. Since both MEK and PI3 kinase signaling pathways are activated in response to DEM treatment [9], we determined whether pharmacological tools that abolish MEK and PI3 kinase activation also affect classical import. For the experiments described here we used kinase inhibitors under conditions that were previously shown to efficiently interfere with Akt and ERK1/2 activation [9]. Thus, LY294002 (LY) abolished the phosphorylation of Akt on Thr308, a process that relies on PI3 kinase activity, whereas PD98059 (PD) drastically reduced the amount of dually phosphorylated ERK1/2, a downstream target of MEK [9].

Incubation of non-stressed or DEM-treated cells with these pharmacological kinase inhibitors led to several significant changes in classical import. First, MEK inhibition stimulated classical import under non-stress conditions. Second, classical import was diminished by DEM; however, this stress-induced inhibition decreased significantly when MEK signaling was abolished (Fig. 1). Together, the results suggest that inhibition of MEK kinases stimulated classical nuclear import, both under normal and oxidative stress conditions.

### Stress, PI3, and MEK Kinase Cascades Control the Localization of Soluble Nuclear Transport Factors and Nucleoporins

**Figure 2:**
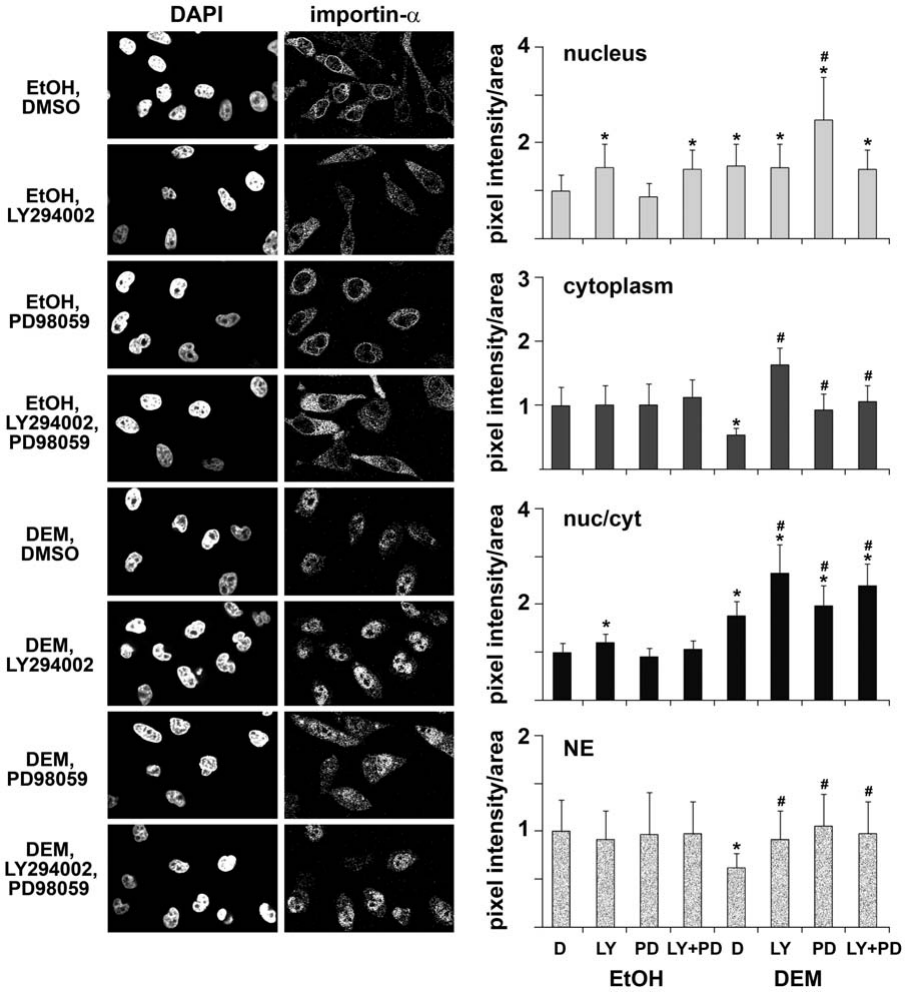
Importin-α localization depends on oxidative stress, PI3 kinase, and MEK signaling. HeLa cells were incubated under the different experimental conditions described for Fig. 1. The intracellular distribution of importin-α was determined by immunofluorescent staining; DAPI was used to visualize nuclei. Nuclear, cytoplasmic and NE fluorescence was measured as described previously [9], [21], [28] and nuc/cyt ratios were calculated. For each compartment pixel intensities/area measured under nonstress control conditions were defined as 1. A minimum of 50 cells was analyzed for each experimental condition. Statistical analyses were performed out as described for Fig. 1.

**Figure 3:**
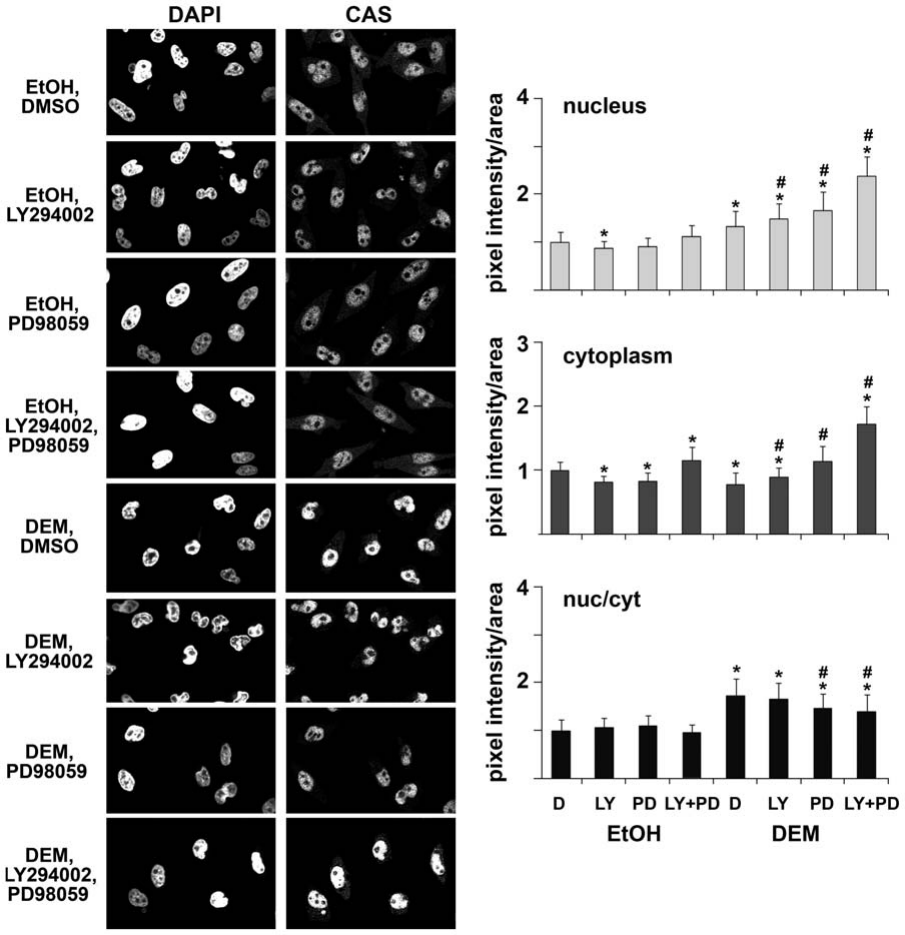
Effect of stress and kinase inhibitors on CAS distribution. Immunofluorescent staining and the quantification of fluorescence intensities was carried out for CAS as described for Fig. 2.

**Figure 4:**
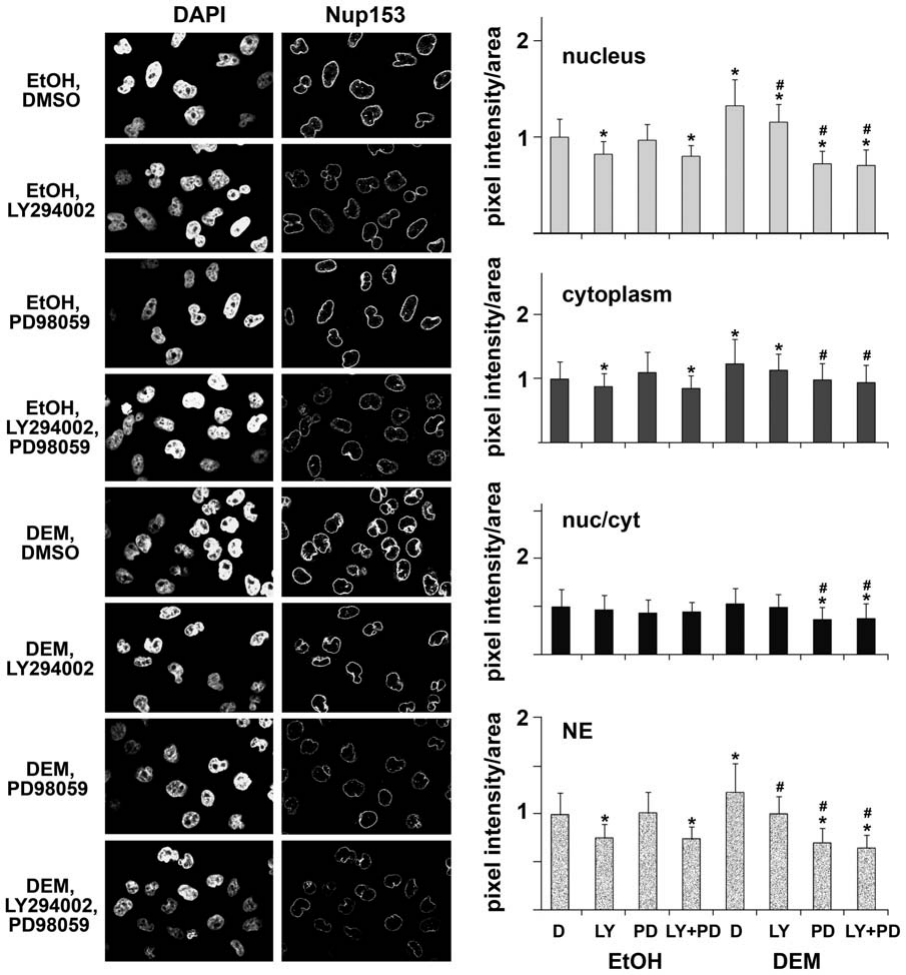
Nup153 distribution upon stress and in the presence of PI3 kinase or MEK inhibitors. Nup153 immunofluorescent staining and the quantification of fluorescence intensities/area were performed as shown for Fig. 2.

**Figure 5:**
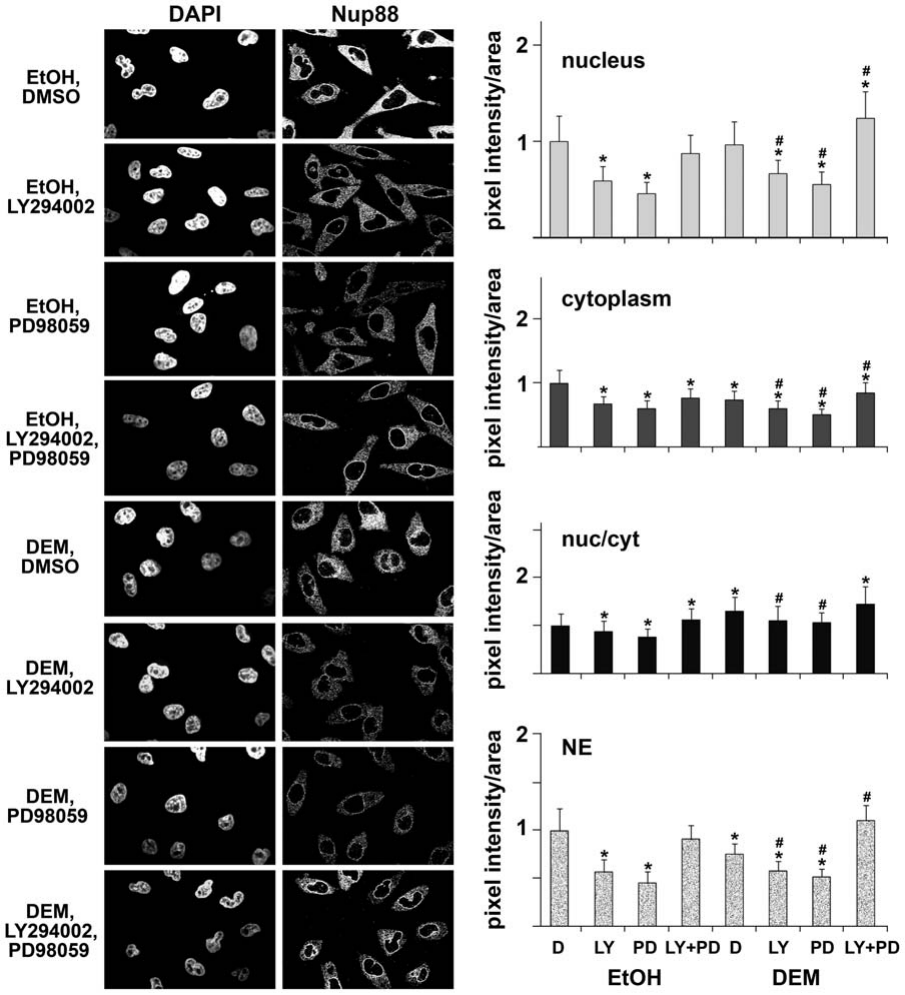
Nup88 localization is sensitive to oxidants and inhibitors of PI3 or MEK kinase signaling. Immunolocalization of Nup88 and measurements of pixel intensities/area were done as detailed for Fig. 2.

For most macromolecules nuclear trafficking is mediated by a specialized transport apparatus that consists of soluble factors and nucleoporins. Many nuclear transport factors reside in multiple cellular compartments, and changes in their steady-state distribution may impinge on nuclear trafficking. Thus, we determined how stress and pharmacological kinase inhibitors altered the localization of transport components. In Figs. 2, 3, 4, and 5 we quantified the concentration of importin-α, CAS, Nup153 and Nup88 in the nuclear interior and in the cytoplasm. We included measurements for the nuclear envelope (NE) association of importin-α, Nup153 and Nup88, since substantial amounts of these proteins reside at the NPC. For each of the cellular compartments, the amount of protein detected under non-stress conditions was defined as 1. [Note that due to the different volumes of nuclei and the cytoplasm an increase in nuclear protein will not necessarily lead to the equivalent decrease in the cytoplasm or at the NE.] As the balance between nuclear and cytoplasmic concentrations of transport factors is likely to impact nuclear trafficking, we further calculated the nuc/cyt ratio; this ratio was defined as 1 for unstressed control cells.

The analyses of four transport components under eight different experimental conditions (s. 2–5) demonstrated that depending on the cellular compartment, stress and kinase inhibitors may have distinct effects. Moreover, individual factors displayed unique profiles of sensitivity towards stress and pharmacological kinase inhibitors. Therefore, in the following sections results are described separately for importin-α, CAS, Nup153 and Nup88. The impact of signaling pathways on classical nuclear import and components of the transport apparatus is summarized in Table 1.

**Table 1:** The activity of signaling pathways correlates with significant changes in classical nuclear import and affects individual transport factors under normal or oxidative stress conditions.

Component analyzed	Signaling pathways with significant impact	
	Control conditions	Oxidative stress
Classical import	MEK	MEK
Importin-α	PI3 kinase	PI3 kinase, MEK
CAS	PI3 kinase	PI3 kinase, MEK
Nup153	PI3 kinase	PI3 kinase, MEK
Nup88	PI3 kinase, MEK	PI3 kinase, MEK

The table summarizes the results obtained for classical nuclear import and the distribution of transport components. Signaling events that lead to significant changes in import or transport factor distribution are listed. See text for details.

### Stress and Signaling Modulate the Concentration of Importin-α in Different Cellular Compartments

Classical nuclear import is mediated by the dimeric carrier importin-α/β1, which requires importin-α as an adaptor. As previous studies did not reveal drastic stress-induced changes in the localization of importin-β1 upon DEM treatment [21], we focused on the analysis of importin-α. Under nonstress conditions, the adaptor is present in the nucleus, cytoplasm and concentrated at the NE (Fig. 2). Following DEM treatment, importin-α concentration in the nucleus and the nuc/cyt ratio increased significantly, whereas the association with NEs was reduced.

The effect of PI3 kinase and MEK inhibitors on importin-α distribution was dependent on the presence of DEM. Without oxidative stress, PI3 kinase and PI3 kinase/MEK inhibition, but not MEK inhibition alone, led to an increase of importin-α in the nucleus. By contrast, in stressed cells PI3 kinase and MEK inhibitors in combination elevated substantially the concentration of importin-α in nuclei (Fig. 2). At the NE and following oxidative stress, the amount of importin-α increased significantly upon PI3 and/or MEK kinase inhibition. Both kinase inhibitors restored importin-α binding to the NE, resulting in amounts that were comparable to unstressed cells.

Taken together, importin-α pools in the nucleus or at the NE responded differently to changes in cell signaling. Without DEM, PI3 kinase inhibition elevated the concentrations of importin-α in nuclei, but not at the NE. In DEM-treated cells, prevention of PI3 and/or MEK kinase activation led to an increase of importin-α both in nuclei and at the NE.

### Stress Upregulates the Nuclear Concentration of CAS, Which Is Further Augmented by PI3 Kinase and MEK Inhibition

Oxidant treatment induced significant changes to CAS concentrations in the nucleus, and it elevated the nuc/cyt ratio of the exporter (Fig. 3). Pharmacological kinase inhibition of unstressed cells led to small changes in the nucleus, whereas the amount of CAS in the cytoplasm was increased when signaling through both PI3 and MEK kinases was prevented. In stressed cells, a pronounced effect of kinase inhibitors was observed, as signals for CAS increased in the nucleus and cytoplasm when PI3 kinase or MEK signaling was blocked. Simultaneous inhibition of PI3 and MEK kinase cascades led to the highest rise in CAS concentrations, both in the nucleus and in the cytoplasm. It is presently not known how inhibition of both PI3 kinase and MEK resulted in such a drastic elevation of CAS amounts in both compartments. This rise may be caused by an increase in CAS synthesis and/or decline in degradation.

Collectively, these data indicate drastic changes for CAS distribution in cells that were treated with oxidant. These changes were even more pronounced when PI3 kinase and/or MEK signaling was abolished.

### Stress Accumulates Nup153 in the Nucleus and at the NE, Which Is Attenuated by the Inhibition of PI3 and MEK Kinase Signaling

The mobile nucleoporin Nup153 shuttles between nuclei and the cytoplasm, at steady-state the protein is concentrated at the nuclear envelope (Fig. 4). Incubation with DEM elevated significantly the amount of Nup153 inside the nucleus, in the cytoplasm and at the NE. This is consistent with our earlier observation [21], which demonstrated that the Nup153 concentration rose in response to oxidant exposure. In all three cellular compartments the stress-induced increase of Nup153 was diminished when signaling through PI3 or MEK kinases was abolished. This reduction was most pronounced when both signaling pathways were inhibited. A different picture emerged in unstressed cells; inhibition of PI3 kinase or of PI3 kinase/MEK combined, but not of MEK alone, reduced significantly the amount of Nup153 in the nucleus, cytoplasm and at the NE.

Taken together, results for Nup153 suggest that under stress conditions both PI3 and MEK kinase cascades contribute to the distribution of Nup153, whereas in unstressed cells PI3 kinase signaling is a key player.

### Nup88 Distribution Changes upon Stress, PI3, and MEK Kinase Signaling Routes Have Different Impact Under Control and Stress Conditions

Like the nuclear transport factors described above, Nup88 displayed a unique profile for the changes in localization that were caused by stress or kinase inhibitors (Fig. 5). The amount of Nup88 was altered in all of the three compartments analyzed here; these changes were most obvious in the nucleus and at the NE.

In the absence of pharmacological drugs, stress diminished the concentration of Nup88 in the cytoplasm and at the NE, leading to an increase in the nuc/cyt ratio. Prevention of PI3 or MEK kinase signaling, either alone or in combination, had similar consequences in control or stressed cells. Specifically, inhibition of the PI3 or MEK signaling pathway reduced the amount of Nup88 in nuclei, the cytoplasm and at the NE, whereas simultaneous interference with both kinase cascades increased drastically the amount of Nup88 in all three compartments. Possible explanations for this rise of Nup88 concentrations in the nucleus, cytoplasm and at the NE under these conditions are changes in the turnover and/or *de novo* synthesis of the nucleoporin. In summary, data obtained with pharmacological compounds suggest that the combined inhibition of PI3 kinase and MEK signaling had the opposite effect when compared to the inhibition of either pathway alone.

### Oxidative Stress Alters the Post-Translational Modification of Importin-α, CAS, Nup153, and Nup88

**Figure 6:**
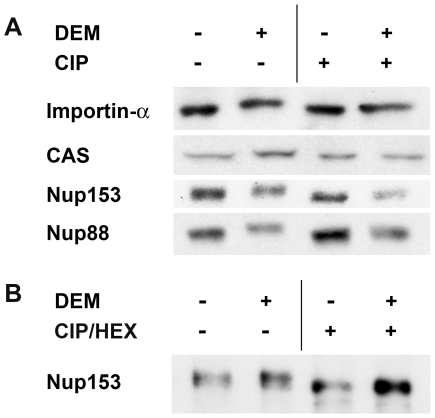
Stress alters the posttranslational modification of transport factors. (A) Phosphorylation contributes to the stress-induced molecular mass changes of nuclear transport factors and nucleoporins. Control and DEM-treated cells were treated with digitonin and detergent-resistant material was incubated in the absence or presence of alkaline phosphatase (CIP). Importin-α, CAS, Nup153 or Nup88 were detected by Western blotting. (B) Crude extracts from unstressed and DEM-treated cells were digested simultaneously with alkaline phosphatase and hexosaminidase (CIP/HEX).

Oxidant exposure may trigger the post-translational modification of proteins, and nuclear transport factors are potential candidates for such modifications. We tested this hypothesis by Western blot analysis of extracts generated from control and stressed cells (Fig. 6A). Side-by-side separation of importin-α, CAS, Nup153 and Nup88 from unstressed and DEM-treated cells revealed that all proteins displayed a slightly higher molecular mass after DEM treatment. To determine the possible contribution of phosphorylation to the increase in apparent molecular mass, proteins were incubated with or without alkaline phosphatase before Western blotting (Fig. 6A). Phosphatase treatment reversed at least in part the stress-induced mobility shift, supporting the idea that oxidative stress triggered the phosphorylation of importin-α, CAS, Nup153 and Nup88.

Several repeat-containing nucleoporins are modified by O-linked glycosylation, and the level of O-GlcNAc modification may increase upon stress [29]. Since treatment with phosphatase did not completely eliminate the stress-induced electrophoretic shift for Nup153, we digested crude extracts with both phosphatase and hexosaminidase. This treatment reversed the stress-dependent increase in molecular mass, suggesting that upon stress both Nup153 phosphorylation and glycosylation are upregulated (Fig. 6B).

**Figure 7:**
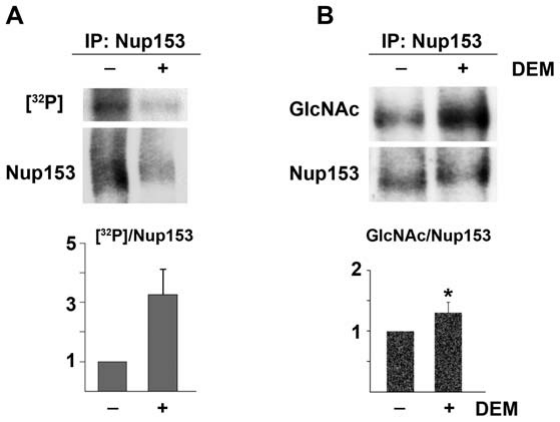
Oxidant treatment increases the phosphorylation and O-glycosylation of Nup153. (A) HeLa cells were incubated with radioactive phosphate under control and stress conditions for 4 hours as described in Materials and Methods. Aliquots of immunopurified Nup153 were separated by SDS-PAGE, and gels were either dried and exposed to film or processed for Western blotting. Signals for radioactivity or ECL were quantified to determine the ratio of [^32^P]/Nup153. Data for unstressed cells were defined as 1. Similar results were obtained for two independent experiments. (B) Nup153 was immunoprecipitated from control and stressed cells under denaturing conditions and further analyzed by Western blotting to detect O-linked glycosylation (GlcNAc) or Nup153. ECL signals were quantified, and the ratio GlcNAc/Nup153 was defined as 1 for control samples. DEM treatment increased this ratio significantly (*P<0.05*). Data are shown for three independent experiments.

We attempted to further test this idea by measuring the incorporation of [^32^P] under normal and stress conditions. To this end, HeLa cells were incubated in the presence of radioactive phosphate, and Nup153 was immunoprecipitated under denaturing conditions. Aliquots of the immunopurified material were separated by SDS-PAGE, and radiolabeling of the immunoprecipitated proteins was measured. In parallel, we determined the amount of immunopurified Nup153 by quantitative Western blotting. From these data, the relative phosphorylation was calculated for Nup153 (Fig. 7A). Results were normalized to control experiments, for which the ratio [^32^P]/Nup153 was defined as 1. With this approach, we detected a 3.3-fold stress-dependent increase in phosphorylation for Nup153.

Additional experiments were carried out to monitor DEM-induced changes in O-glycosylation of Nup153. The nucleoporin was immunoprecipitated under denaturing conditions and subjected to Western blotting with antibodies recognizing O-GlcNAc modifications or Nup153. Results depicted in Fig. 7B demonstrate that DEM treatment caused a 1.3-fold increase in Nup153 O-glycosylation; albeit small, this increase was statistically significant.

**Figure 8:**
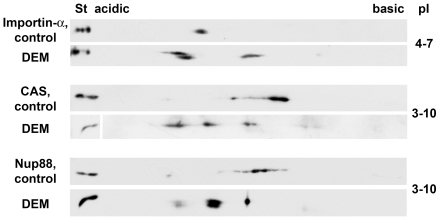
Oxidative stress alters the isoelectric points of importin-α, CAS, and Nup88. HeLa cells were incubated with the solvent ethanol or DEM, and crude extracts were separated by 2D electrophoresis and Western blotting. Isoelectric focusing was performed with pH gradients of 4–7 or 3–10 NL (non-linear) as indicated in the figure. As reference, aliquots of the starting material were run on the same gel (St). Results are representative of four independent experiments. For all transport factors analyzed, at least a portion became more acidic after DEM treatment.

Under the conditions used here for radiolabeling, the incorporation of [^32^P] was low or not detectable for importin-α, CAS and Nup88, both in control and stressed cells. Thus, this method could not be applied to detect changes in phosphorylation. We circumvented this complication by performing 2D gel electrophoresis combined with Western blotting in order to monitor possible changes to the isoelectric points of transport components. Data in Fig. 8 show the staining pattern obtained when samples from control and oxidant-treated cells were analyzed side-by-side. For all proteins examined, the distribution of spots was altered by DEM incubation, and a portion of importin-α, CAS and Nup88 displayed more acidic isoelectric points in response to stress. These results are consistent with the model that for a subpopulation of transport factors phosphorylation increased as a result of oxidant exposure. It is not known at this point why a fraction of importin-α became more basic in DEM-treated cells.

Together, data in Figs. 6, 7, and 8 are in line with the idea that the extent of post-translational modification of several transport factors depends on the physiological state of the cell, as it is sensitive to stress. These modifications include phosphorylation and O-linked glycosylation.

## Discussion

With the present study we examined in a quantitative fashion the link between oxidative stress, intracellular signaling and the nuclear transport apparatus. Our results show that the activation of MEK and PI3 kinase cascades provides a novel mechanism to govern the distribution of multiple nuclear transport factors. Moreover, we demonstrate that oxidant treatment not only relocated importin-α, CAS, Nup153 and Nup88, it also altered their post-translational modification.

Interestingly, individual transport factors displayed unique profiles when their distribution was analyzed in response to stress or changes in signaling. Results for importin-α and CAS are of particular interest with respect to classical nuclear import. Both transport factors are required for nuclear trafficking of numerous cargos, either directly as a subunit of the classical NLS-receptor or indirectly by recycling importin-α to the cytoplasm. Differences in the localization and modification of importin-α or CAS are likely to affect all of the cargos, and these changes are likely to be relevant to cell physiology. As such, transport of proteins into the nucleus is an essential step of the stress response and necessary to cope with stress-induced damage. Transcription factors play a crucial role in the response to oxidant exposure, as they control the expression of genes encoding antiapoptotic proteins or detoxifying enzymes. Prominent examples that are relevant to oxidative stress include NF-κB, Nrf2, CREB and p53, all of which rely on importin-α isoforms for translocation across the NE [30]–[36]. Importantly, mislocalization of these transcriptional regulators has been linked to neurodegenerative diseases [37], and it is feasible that their delivery to the nucleus is regulated by the steady-state distribution of importin-α or CAS.

In previous studies, we analyzed how oxidative stress affects the activation and localization of ERK1/2 and Akt kinases, key components of MEK and PI3 signaling cascades. This research demonstrated crosstalk between MEK and PI3 kinase pathways, and identified a novel signaling link in the nucleus of stressed cells that connects Akt phosphorylated on Ser473 with dually phosphorylated ERK1/2 [9]. With respect to our present contribution, it is of interest that a similar profile was observed for changes in the nuclear distribution of importin-α, p-ERK1/2 and p-Akt473 (Fig. S1). Several importin-α sites have been shown to be phosphorylated [38], and database searches predict sites that are potentially modified by Akt or ERK1/2 [39]. It will be interesting to determine in future experiments whether importin-α is a substrate for either of these kinases.

Despite the crucial roles importin-α and CAS play in nuclear transport, other cellular functions have been described for both proteins which are likely to depend on their proper intracellular distribution [16], [17], [40]. Thus, the role of CAS in p53-mediated gene expression and its link to apoptosis indicate that multiple aspects of cell physiology will be modulated when CAS accumulates in nuclei.

Previous results by others suggested that in unstressed cells MEK1 controls CAS localization, as treatment with PD98059 was reported to increase the concentration of CAS in nuclei [41]. In our studies, we did not observe a significant change in nuclear CAS when MEK was inhibited under nonstress conditions. However, for the experiments described here the amount of CAS increased in DEM-treated cells, and this was further augmented by MEK inhibition. The reasons for the different effects of MEK inhibition on CAS localization are currently not clear. One possibility is that the high density of cells used in previous studies [41] may alter intracellular signaling and the localization of transport factors. Our earlier studies demonstrated the impact of cell density on the nucleocytoplasmic distribution of proteins [42 and data not shown], which could also apply to CAS.

Like importin-α and CAS, the stress-induced changes observed for Nup153 and Nup88 are likely to have broad implications for nuclear transport and organization. As such, Nup153 is not only involved in classical nuclear import, it also participates in other nuclear protein transport pathways and RNA trafficking. In addition, Nup153 mobility depends on the transcriptional activity of cells [43], a process likely to be sensitive to oxidative stress and cell signaling events. Thus, it is reasonable to speculate that changes in Nup153 localization affect several transport routes that move protein and RNA across the NE. The same applies to Nup88, a structural component of NPCs that contributes to nuclear transport. Moreover, Nup88 functions are not limited to trafficking across the NE, as more recent studies revealed that inside the nucleus Nup88 participates in the regulation of transcription [22].

The nuclear accumulation of many proteins and especially a large number of transcription factors is regulated by signaling through PI3 kinase and MEK-dependent pathways. Data presented here, however, suggest that not only individual cargos, but nuclear transport in general is linked to these signaling events. Interestingly, our data show that the effect of signaling is different under normal and stress conditions. This scenario is further complicated by the fact that the impact of stress and signaling may be unique for a particular cellular compartment. Thus, we demonstrate here that intranuclear, NE and cytoplasmic pools of transport factors can respond differently to changes in cell physiology. Ultimately, these compartment-specific changes may be the combination of several not mutually exclusive mechanisms, which could include changes in nuclear import, cytoplasmic and nuclear retention, or export to the cytoplasm.

Another layer of complexity may be added by stress- or signaling-induced differences in transport factor concentration. For instance, the overall amount of Nup153 [21] and possibly CAS (this contribution) could be altered when cell physiology changes. Although CAS concentrations are not limiting for nuclear transport under normal conditions [44], stressed cells may have different requirements, both for nuclear trafficking and unrelated functions.

An alternative explanation for the effects elicited by DEM is a direct reaction of the chemical with cellular components [45], which could ultimately alter nuclear transport. Our studies do not exclude the possibility that DEM modifies nuclear transport factors, and this process may in part contribute to the DEM-induced changes observed by us. If such covalent modifications occur, however, it is difficult to envision how they are modulated by cell signaling.

It should be noted that our studies did not determine whether treatment-dependent differences in cell volume contribute to the changes in transport factor concentration in the nucleus, cytoplasm or at the NE. Future experiments will have to address these questions by measuring volumes and voxel intensities for individual compartments.

In addition to the complex effect of stress and signaling on transport factor distribution, we demonstrate that their post-translational modification is sensitive to oxidant exposure. Transport factor phosphorylation is likely to be dynamic, with protein kinases and phosphatases determining the steady-state levels of modification. At present, kinases and phosphatases involved in the stress-induced modification of importin-α, CAS, Nup153 and Nup88 have yet to be identified. Nevertheless, it is noteworthy that upon oxidant exposure multiple kinase cascades, including PI3 and MEK-dependent routes, are activated, whereas several protein tyrosine and lipid phosphatases are transiently inactivated [5], [9], [46]. Collectively, this suggests that changes in the redox state will alter kinase and/or phosphatase activity, thereby increasing the concentration of phosphorylated transport factors and possibly altering their function. The same reasoning applies to the O-glycosylation for Nup153. It has been established that the stress-induced increase in O-GlcNAc modifications improves cell survival [29], and it is possible that Nup153 glycosylation plays a pivotal role in this process.

During the revision of this manuscript, it was shown that under nonstress conditions ERK1/2 activity is required to phosphorylate two sites in Nup153 [47]. Furthermore, pulldown experiments revealed that Nup153 phosphorylation by ERK1/2 *in vitro* prevents importin-β1 binding. We have demonstrated earlier that DEM-treatment activates ERK1/2 and increases the concentration of dually phosphorylated ERK1/2 in nuclei [9]. Taken together, these results could indicate that the DEM-induced increase in Nup153 phosphorylation is caused by ERK1/2 activation. If this is the case, Nup153 phosphorylation in response to DEM incubation may reduce its association with import complexes or cargo-free importin-β1. Ultimately, this could contribute to a decrease in classical nuclear import.

Although results presented here are consistent with stress-induced changes in the phosphorylation of multiple transport factors, the evidence is at present only indirect for importin-α, CAS and Nup88. As such, our experiments demonstrate that the isoelectric points of these proteins are altered in response to stress. Notably, some but not all of the shifts are consistent with an oxidant-dependent increase in phosphorylation. Moreover, the complex pattern obtained in these experiments may indicate that in stressed cells transport factors are subject to other modifications as well.

Knowledge about the signaling events that control the nuclear transport apparatus under normal and stress conditions is only beginning to emerge [47], [48]. As such, RanBP3 was identified as a downstream target of ERK/RSK and PI3K/Akt signaling [48], with both pathways regulating RanBP3 phosphorylation. *In vitro*, the loss of RanBP3 phosphorylation impinges on RCC1 function by reducing RCC1-mediated guanine nucleotide exchange on Ran. In growing cells, prevention of RanBP3 phosphorylation altered the nucleocytoplasmic Ran gradient, ultimately compromising nuclear protein import. Whether RanBP3 localization was modulated by signaling or other changes in cell physiology, however, was not investigated [48]. Interestingly, the stress conditions employed in our experiments did not induce a collapse of the Ran gradient [21], and no drastic changes in RanBP3 localization were detected for DEM-treated cells [unpublished data by N. Crampton and U. Stochaj]. Taken together, these results suggest that nuclear transport factors other than RanBP3 are the critical components that control classical import under the stress conditions analyzed by us.

**Figure 9:**
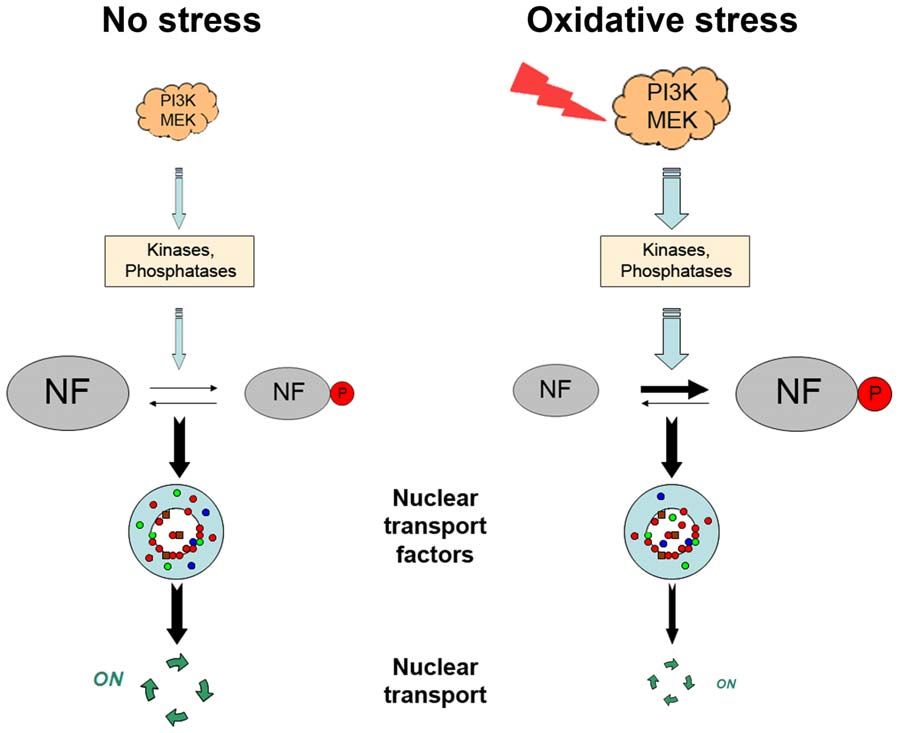
Classical nuclear import is regulated by signaling and stress. A simplified model depicts the possible role of signaling and oxidative stress in the control of classical import. Relocation and changes in post-translational modification of multiple nuclear transport factors (NF) correlate with a reduction in classical nuclear import.

A simplified model depicted in Fig. 9 summarizes the results described here. With respect to nuclear transport and other processes that rely on importin-α, CAS, Nup153 or Nup88, we speculate that the proper localization and post-translational modification will contribute to the activity of each component. This includes, but is not limited to, their function in nuclear transport. The data presented here provide a basis to explore these different avenues and define the events that regulate individual transport factors and their functions in distinct cellular compartments.

Taken together, our analyses in growing cells revealed that under nonstress conditions PI3 kinase had the most prominent effect on transport factors (Table 1), whereas classical import was regulated by MEK-dependent signaling. This is in line with *in vitro* nuclear import experiments that suggested a role for ERK2 in nuclear transport [24]. A different scenario, however, emerged in stressed cells. Following DEM treatment, MEK signaling was crucial for classical import inhibition, whereas both MEK and PI3 kinase played a significant role in transport factor relocation (Table 1). Thus, different profiles were obtained for classical import and individual transport factors when their sensitivity to stress or kinase inhibitors was examined. This outcome supports our hypothesis that the combination of changes affecting multiple transport factors, including but not limited to those analyzed by us, will determine the ultimate consequences for nuclear trafficking.

## Materials and Methods

### Growth and Stress Exposure of HeLa Cells

HeLa cells were grown in multiwell chambers to 50 to 70% confluency [9] and subjected to incubation with DEM or the solvent ethanol as indicated. Cells were preincubated for 1 h with 25 µM PD98509 or 50 µM LY294002 dissolved in DMSO or solvent immediately before treatment with DEM for 4 hours at 37°C [9]. Inhibitors or DMSO were present throughout the incubation with DEM.

### Immunofluorescent Staining and Quantification of Protein Localization

Protocols for immunofluorescent staining, confocal microscopy, image acquisition and analysis are detailed in [9], [21], [28].

### Statistics

To measure fluorescence signals in nuclei and cytoplasm, at least 50 cells were scanned by confocal microscopy for each of the different conditions. For quantification, fluorescence signals were integrated over the entire nucleus, cytoplasm or NE. Data were acquired for at least three independent experiments. Results are shown as means±STDEV; One-way ANOVA was carried out to identify significant differences.

### Western Blot Analysis

HeLa cells were grown on dishes to 50 to 70% confluency. Cells were stressed as described above, washed with PBS and stored at −70°C until use. Crude extracts were prepared by solubilizing proteins in gel sample buffer, pH 8.0, containing protease inhibitors (1 mM PMSF; aprotinin, leupeptin, pepstatin, each at 1 µg/ml), 20 mM β-glycerophosphate, 1 mM NaN_3_, 2.5 mM NaF. Samples were incubated for 10 min at 95°C and vortexed with glass beads to shear DNA. After centrifugation (5 min, 13,000 rpm, microfuge) equal amounts of protein were separated in SDS-PA gels. Proteins were blotted to nitrocellulose and blots processed as described [26]. Antibodies were used at the following dilutions: Nup153, (1∶200); Nup88 (1∶1,000; BD Biosciences; Mississauga, ON); O-linked N-Acetylglucosamine (1∶2,000; ABR, MA1-072). All other antibodies were purchased from Santa Cruz Biotechnology: importin-α1 (1∶500, sc-6917), CAS (1∶200, sc-1708).

### Phosphatase Treatment

HeLa cells were incubated with ethanol or 2 mM DEM for 4 hours, extracted with digitonin to remove cytoplasmic proteins and boiled in 40 mM Tris HCl pH 8.0, 1% SDS, 50 mM DTT containing 1 mM NaN_3_, 2.5 mM NaF and a cocktail of protease inhibitors (aprotinin, pepstatin, leupeptin, each at 1 µg/ml). DNA was sheared by vortexing with glass beads during the incubation period and insoluble material was removed by centrifugation (5 min, 13,000 rpm). Supernatants were diluted 1∶2 in water and precipitated with 10% TCA for 1 h on ice. Sediments were resuspended in 100 mM Tris HCl pH 7.5, 50 mM NaCl, 10 mM MgCl_2_, 0.025% Triton X-100 and incubated with 200 U/ml calf intestinal phosphatase (CIP, New England Biolabs, Pickering, ON) for 1 h at 37°C. Controls were incubated under identical conditions without adding CIP.

### Incubation with Hexosaminidase and CIP

Treatment with hexosaminidase essentially followed published procedures [49]. Briefly, samples were scraped into 1% SDS, boiled for 5 min, vortexed with glass beads and centrifuged 5 min at 13,000 rpm (microfuge). Supernatants were diluted 1∶2 into 100 mM sodium citrate pH 6.7, 8% Triton X-100, 20 mM MgCl_2_, 2 mM DTT, and twofold concentrated protease inhibitors. Samples were incubated for 20 h with 100 U/ml hexosaminidase and 200 U/ml CIP and subjected to Western blotting. Hexosaminidase was sufficiently active under these conditions to remove GlcNAc moieties from proteins (Fig. S2).

### In Vivo Labeling with [^32^P] Phosphate and Immunoprecipitation under Denaturing Conditions

HeLa cells were labeled with [^32^P] phosphate essentially as described [50]. In brief, cells were incubated with radioactivity for 4 hours at 37°C under control or stress conditions; serum was present throughout the incubation period. After removal of the medium samples were washed with 150 mM NaCl, 50 mM Tris, pH 7.4 and stored at −70°C. Crude extracts prepared in 50 mM Tris pH 6.8, 1% SDS, 5 mM DTT were heated for 10 min at 95°C and vortexed with glass beads. Extracts were centrifuged for 5 min at 13,000 rpm (microfuge) and supernatants diluted 1∶10 into PBS, 1% NP-40, 1 mM NaN_3_, 2.5 mM NaF, 20 mM β-glycerophosphate, 1 mM sodium orthovanadate and protease inhibitors (Roche). Samples were pre-adsorbed with Protein G-Plus agarose (Santa Cruz Biotechnology) for 30 min at 4°C and beads were removed by centrifugation. After 1 hour incubation with antibodies at 4°C, Protein G-Plus agarose was added and samples were kept overnight at 4°C. Beads were collected by centrifugation and washed three times with PBS/1 mM NaN_3_. Bound material was eluted by incubating in twofold concentrated gel sample buffer for 10 min at 95°C. Resin was removed by centrifugation and supernatants were subjected to SDS-PAGE and Western blotting.

### 2D Gel Electrophoresis

HeLa cells treated with ethanol or DEM were rinsed with PBS and frozen at −70°C. Samples were resuspended in 7 M urea, 2 M thiourea, 4% (w/v) CHAPS, 30 mM Tris, 20 mM DTT, 1% Bio-Lyte 3–10 (Bio-Rad), vortexed with beads to shear DNA and centrifuged for 5 min at 13,000 rpm (microfuge). Supernatants were applied to 7 cm IPG strips 4–7 or 3–10 NL (non linear gradient), all purchased from Bio-Rad. After 14 h of rehydration, focusing was carried out with a Protean IEF cell (Bio-Rad) as recommended by the manufacturer. Strips were either immediately processed for the second dimension or frozen at −70°C. SDS-PAGE and Western blot analysis followed the procedures described above.

## Supporting Information

Figure S1 Effect of pharmacological kinase inhibitors and stress on importin-α, dually phosphorylated ERK1/2 (p-ERK1/2), and Akt phosphorylated on Ser473 (p-Akt473) in nuclei. Changes in the nuclear concentration of importin-α (shown in Fig. 2) are compared to the distribution of p-ERK1/2 and p-Akt473 [9]. For each set, results were normalized to unstressed cells incubated with DMSO (D, EtOH). Data for p-ERK1/2 and p-Akt473 were reproduced from [9] with permission.
(8.82 MB TIF)

Figure S2 Hexosaminidase is active under conditions used for hexosaminidase and CIP double digests. Samples were probed with antibodies against O-GlcNAc to monitor the activity of hexosaminidase under the conditions used for Fig. 6B. For samples incubated with hexosaminidase and CIP, binding of O-GlcNAc-specific antibodies was no longer detected.
(3.67 MB TIF)
