# Supplementary figures and images for: Dissecting the Signaling Events That Impact Classical Nuclear Import and Target Nuclear Transport Factors
Source: PLoS One. 2009 Dec 24;4(12):e8420. doi: 10.1371/journal.pone.0008420 (PMC2793512; doi:10.1371/journal.pone.0008420)

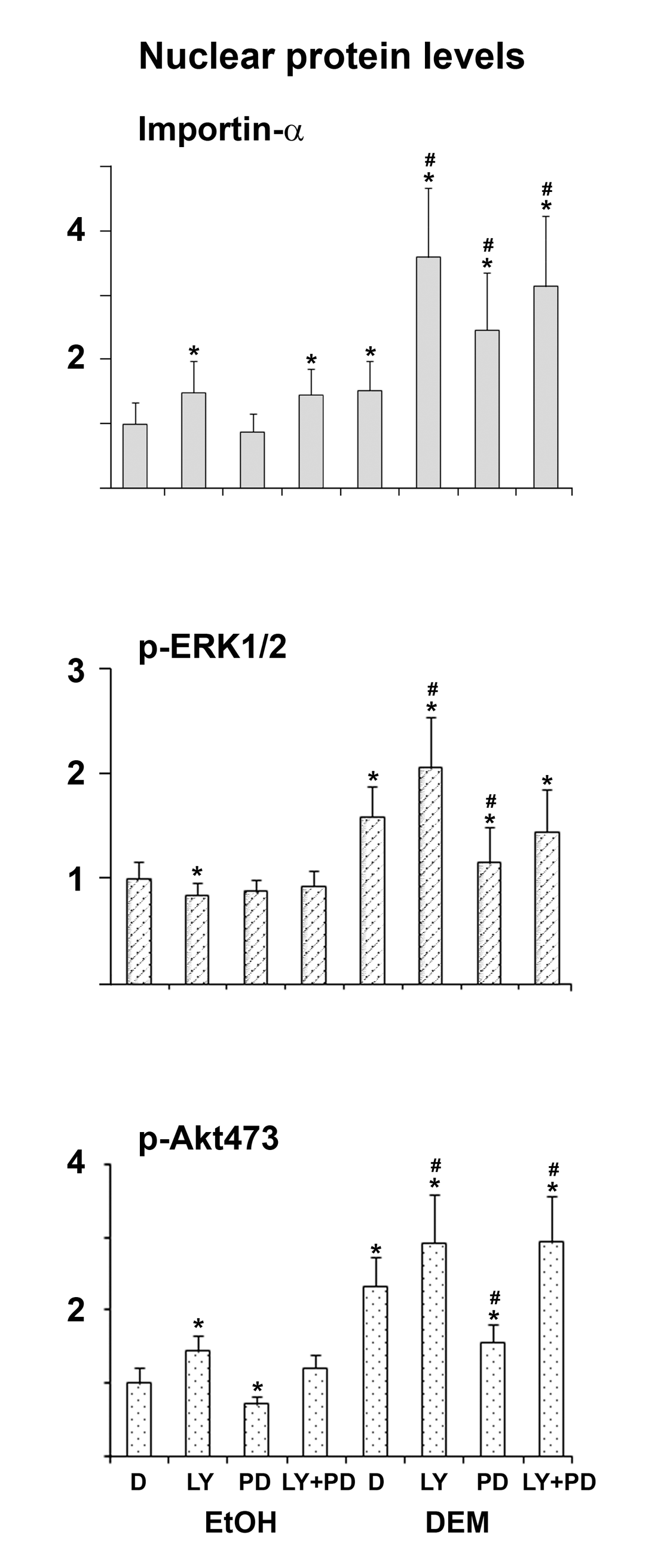

Supplement: Figure S1 — Effect of pharmacological kinase inhibitors and stress on importin-α, dually phosphorylated ERK1/2 (p-ERK1/2), and Akt phosphorylated on Ser473 (p-Akt473) in nuclei. Changes in the nuclear concentration of importin-α (shown in Fig. 2) are compared to the distribution of p-ERK1/2 and p-Akt473 [9]. For each set, results were normalized to unstressed cells incubated with DMSO (D, EtOH). Data for p-ERK1/2 and p-Akt473 were reproduced from [9] with permission. (8.82 MB TIF) [file pone.0008420.s001.tif]

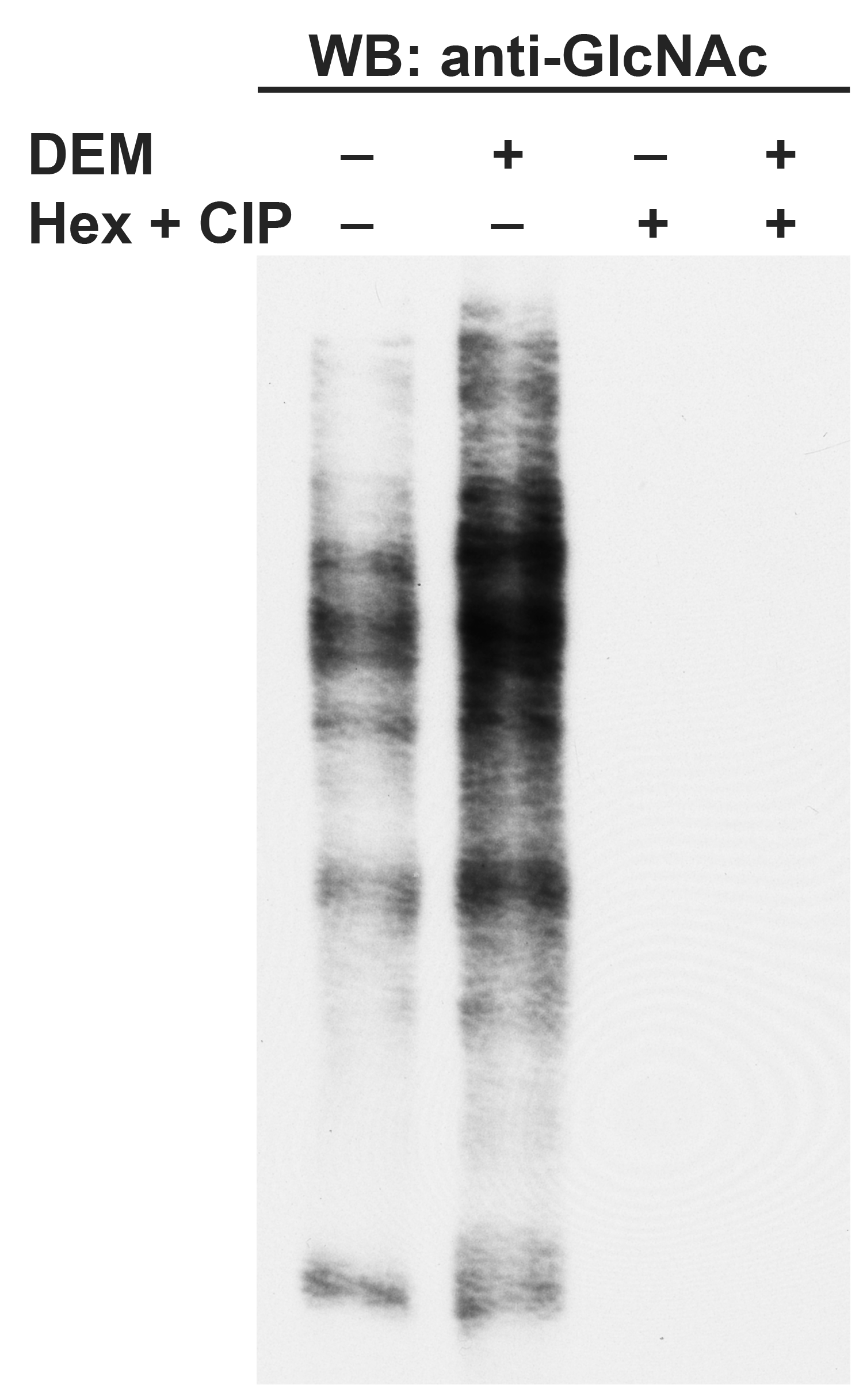

Supplement: Figure S2 — Hexosaminidase is active under conditions used for hexosaminidase and CIP double digests. Samples were probed with antibodies against O-GlcNAc to monitor the activity of hexosaminidase under the conditions used for Fig. 6B. For samples incubated with hexosaminidase and CIP, binding of O-GlcNAc-specific antibodies was no longer detected. (3.67 MB TIF) [file pone.0008420.s002.tif]
